# Relationship Between Visceral Metastases and Survival in Patients with Metastasis‐related Spinal Cord Compression

**DOI:** 10.1111/os.12465

**Published:** 2019-04-15

**Authors:** Deng‐xing Lun, Xiao‐dong Wang, Yu‐dong Ji, Yong‐cheng Hu, Xiong‐gang Yang, Xiu‐chun Yu, Guo‐chuan Zhang, Qing‐shan Zhuang

**Affiliations:** ^1^ Department of Spine Surgery Weifang People's Hospital Weifang Shandong China; ^2^ Tianjin Traditional Chinese Medicine University Tianjin China; ^3^ Department of Orthopaedic Sunshine Union Hospital Weifang Shandong China; ^4^ Department of Bone Oncology Tianjin Hospital Tianjin China; ^5^ Department of Orthopaedic Oncology Jinan Military General Hospital Jinan China; ^6^ Department of Orthopaedic Surgery Third Hospital of Hebei Medical University Shijiazhuang Shi Hebei Sheng China

**Keywords:** Karnofsky performance status, Metastasis‐related spinal cord compression, Overall survival, Primary tumor, Prognostic factors

## Abstract

**Objective:**

To investigate whether visceral metastases have a significant impact on survival in patients with metastasis‐related spinal cord compression (MSCC), and to determine the difference in prognosis between patients with and without visceral metastases.

**Methods:**

Three institutional databases were searched to identify all patients who had undergone spinal surgery for spinal metastases between March 2002 and June 2010. Data on patient characteristics including pre‐ and post‐operative medical conditions, were collected from medical records or by telephone follow‐up. Survival data were obtained either from medical records or by searching a governmental cancer registry.

**Results:**

The mean age of study patients was 59.6 ± 10.5 years (range, 18–84 years), of whom 102 were male and 67 female. The median and mean postoperative survival times were 7.0 ± 0.5 (95% CI 6.0–8.0) months and 12.6 ± 1.2 (95% CI 10.1–15.0) months, respectively, in all patients, being 5.0 ± 0.5 (95% CI 4.0–6.0) months and 10.8 ± 2.4 (95% CI 6.1–15.5) months, respectively, for patients with visceral metastases and 7.0 ± 0.8 (95% CI 5.4–8.6) months and 13.0 ± 1.4 (95%CI 10.3–15.6) months, respectively, for patients without visceral metastases (*P* = 0.87). These survival times did not differ significantly between groups. Multivariate Cox proportional hazard regressions showed that visceral metastases had no statistically significant association with survival (*P* = 0.277), whereas rate of growth of primary tumor (*P* = 0.003), preoperative Karnofsky performance status (KPS) (*P* < 0.001), change in KPS (*P* < 0.001), and Frankel grade (*P* = 0.091) were independent prognostic factors in the whole cohort (*P* = 0.005). Changes in KPS (*P* = 0.001) and major complications (*P* = 0.003) were significantly associated with survival in patients with visceral metastases, whereas rate of growth of primary tumor (*P* = 0.016), change in KPS (*P* = 0.001), and preoperative KPS (*P* < 0.001) were significantly associated with survival in patients without visceral metastases.

**Conclusions:**

Visceral metastases do not appear to predict the prognosis of patients with MSCC; thus, more aggressive surgery should be considered in patients with MSCC who have visceral metastases. Additionally, prognostic factors differ according to visceral metastases status in these patients.

## Introduction

Recent treatment regimens have prolonged median survival time in patients with cancer, which has consequently led to a high frequency of metastatic spinal cord compression (MSCC) during the remaining lifetime of these patients. Approximately, 70% of patients with cancer develop spinal metastases^1^, ^2^, 20% of whom develop neurological deficits^3^, ^4^, ^5^. Almost 10% of patients with MSCC choose to undergo surgical decompression with or without stabilization^4^, ^6^, ^7^, ^8^, which can restore neurological function and improve their quality of life. However, it is not yet clear how to identify the patients who would benefit most from surgical treatment. It is generally accepted that life expectancy drives treatment regimens for spine metastases^9^. For example, decompressive surgery is generally not considered indicated in patients with life expectancies of less than 3 months^10^.

Some surgeons and radiologists have therefore established various prognostic scoring systems for predicting survival to help decide in selection of the most appropriate treatment strategy^11^. Unsurprisingly, because visceral metastases are considered to indicate the terminal stage in patients with cancer and their treatment is palliative rather than curative, visceral metastases have been regarded as one of the most important, and therefore commonly used, prognostic factors. This factor has therefore been incorporated into all of these scoring systems^12^, ^13^, ^14^, ^15^, ^16^, ^17^, ^18^, ^19^.

However, recently published studies have reported significantly disparate findings concerning the effect of visceral metastases on survival. Arrigo *et al.* 20 reported that visceral metastases do not significantly influence survival after surgery in patients with MSCC. Chong *et al.* 21 investigated preoperative prognostic factors in 108 patients and showed that visceral metastases are not an independent prognostic factor despite the median survival of patients with visceral metastases at the time of surgery being 4.0 months and that of patients without visceral metastases 11.0 months. Therefore, there is controversy over whether visceral metastases are a prognostic factor in patients with spinal metastases.

The current study was performed with the goals of further identifying the role of visceral metastases in predicting survival time in patients with spinal metastases and determining the difference in prognosis between patients with and without visceral metastases.

## Methods

This study was approved by the hospital Ethics Committee. Three institutional databases were searched to identify all patients with spinal metastases and Tokuhashi score 9–15 between March 2002 and June 2010^14^.

The inclusion criteria for performing surgical interventions comprised intractable pain despite medication, rapidly progressive neurological deterioration, and evidence of clinical or radiographic instability.

The exclusion criteria comprised spinal metastases without cord compression, treatment by radiotherapy or revision procedures, operative procedure vertebroplasty or kyphoplasty only, life expectancy less than 3 months, and patients whose medical condition was considered too poor to tolerate surgery. Life expectancy was estimated on the basis of the revised Tokuhashi scoring system. Additionally, surgery was selected by mutual agreement between the surgeon and patient.

Survival data were obtained from medical records, by telephone follow‐up, or searching a governmental cancer registry. The patients were divided into two groups according to whether they had visceral metastases. Patient characteristics, including preoperative and postoperative medical conditions, were collected from medical records or by telephone follow‐up. Selected possible prognostic factors were analyzed, and each variable was categorized into two or three groups as follows: age (<65 vs. ≥65 years), sex (female vs. male), rate of growth of primary tumor (rapid vs. moderate vs. slow), preoperative and postoperative Frankel scores (A–C vs. D–E), other bone metastases (no vs. yes), preoperative and postoperative Karnofsky performance status (KPS) (10–40 vs. 50–70 vs. 80–100), number of involved vertebrae (solitary vs. multiple), pathological fracture (no vs. yes), metastasis site (cervical vs. non‐cervical), serum albumin concentration(<35 g/L vs. ≥35 g/L), sphincter dysfunction (no vs. yes), and interval between developing motor deficits and surgery (≤5 vs. >5 days).

On the basis of findings reported by Tomita^12^, primary cancer types were categorized according to growth rate as follows: slow growth (breast, prostate, thyroid, etc.), moderate growth (kidney, uterus, etc.) and rapid growth (lung, colon, liver, gastric cancer, and other cancers).

Postoperative survival was defined as the time between the date of surgery and death or the latest follow‐up. Neurological function was graded according to Frankel grade preoperatively and 4 weeks postoperatively (patients with Frankel D and E are able to walk). Time to developing motor deficits was defined as interval between deterioration of motor function and surgery. Deterioration of motor function was defined as a change of at least one Frankel grade.

### *Statistical Analysis*

Mean values are reported as mean ± standard deviation and median values with range. The characteristics of the two groups were compared using the χ^2^ or Student's *t‐*test, and a two‐tailed *P* <0.05 was considered to denote statistical significance. Univariate analysis of survival was performed using the Kaplan–Meier method and log‐rank test. Variables significant at *P* < 0.01 in the univariate analysis were tested through a backward stepwise selection process for their independent effect on overall survival (OS). Rate ratios and their 95% confidence intervals (CIs) were computed, as were odds ratios and their 95% CIs. *P* < 0.05 was considered to denote statistical significance.

## Results

### *Patient Characteristics*

Patient characteristic according to group are summarized in Table 1. There were 102 men and 67 women with a mean age of 59.6 ± 10.5 years (range, 18–84 years). Forty‐two patients had visceral metastases at the time of spinal surgery and 127 did not. The primary cancers were lung cancer (73 patients, 43%), breast cancer (13, 8%), renal cancer (12, 7%), hepatic cancer (10, 6%), gastrointestinal cancer (nine, 5%), prostate cancer (seven, 4%), and other (45, 27%).

**Table 1:** Baseline characteristics of the study cohort and patient subgroups

Variables	All patients	Patients with visceral metastasis	Patients without visceral metastasis	*P* value
Number of patients	169	42	127	—
Age(mean ± SD)	59.6 ± 10.5	60.3 ± 11.4	59.4 ± 10.3	0.733
Age‐N (%)				0.477
<65	109	29	80	
≥65	60	13	47	
Gender‐N (%)				0.423
Male	102	23	79	
Female	67	19	48	
Systematic co‐morbidity‐N (%)				0.729
Yes	56	13	43	
No	113	29	84	
Type of primary tumor‐N (%)				0.136
Group A (rapid)	78	14	64	
Group B (moderate)	65	19	46	
Group C (slow)	26	9	17	
Location of involved vertebrae‐N (%)				0.778
Cervical	22	6	16	
Non‐cervical	147	36	111	
Frankel grade pre‐operation‐N (%)				0.437
A‐C	37	11	26	
D‐E	132	31	101	
Extrospinal bone metastasis‐N (%)				0.229
Yes	113	22	53	
No	56	20	74	
Pathological fracture‐N (%)				0.720
Yes	33	9	24	
No	136	33	103	
Number of involved vertebrae‐N(%)				0.826
Yes	41	20	58	
No	128	22	69	
Preoperative KPS‐N (%)				**0.044** *
10–40	19	8	11	
50–70	94	17	77	
80–100	56	17	39	
Time to developing motor deficit‐N (%)				0.446
≤5 days	121	32	89	
>5 days	48	10	38	
Urinary retention/incontinence‐N (%)				0.877
Yes	13	3	10	
No	156	39	117	
Serum album level (g/l)‐N (%)				**<0.001** *
<35g/l	17	10	7	
≥35g/l	96	18	78	
Adjuvant therapy‐N (%)				0.304
Yes	125	24	101	
No	44	4	40	
Local relapse after treatment‐N (%)				0.375
Yes	32	6	26	
No	137	36	101	
Major complications post‐operation‐N (%)				0.649
Yes	15	3	12	
No	154	39	115	
Change on Frankel grade‐N (%)				0.414
Deteriorated	15	2	13	
Not changed	75	24	51	
Improved	79	16	63	
Change on Karnofsky performance score‐N (%)				0.306
Deteriorated	22	6	16	
Not changed	46	14	32	
Improved	101	22	79	

Note: *, statistical significance; N, number.

**Figure 1:**
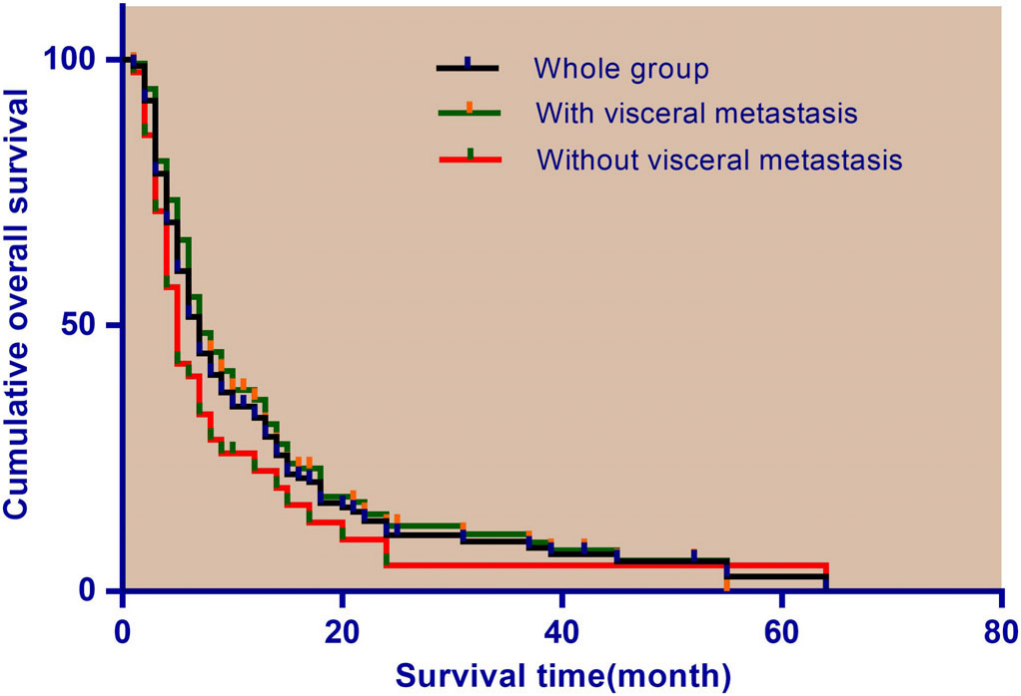
Kaplan–Meier survival analysis for whole cohort and subgroups with and without visceral metastases.

The median and mean postoperative survival times were 7.0 ± 0.5 (95%CI 6.0–8.0) months and 12.6 ± 1.2 (95%CI 10.1–15.0) months, respectively, in the whole cohort, being 5.0 ± 0.5 (95%CI 4.0–6.0) months and 10.8 ± 2.4 (95%CI 6.1–15.5) months, respectively, in patients with visceral metastases and 7.0 ± 0.8 (95% CI 5.4–8.6) months and 13.0 ± 1.4 (95% CI 10.3–15.6) months, respectively, in patients without visceral metastases (*P* = 0.87) (Fig. 1). There was a trend toward lower OS rates in patients with visceral metastases compared with those without them; however, this difference was not significant (HR 1.28, 95% CI 0.82–2.01, *P* = 0.277). The 6‐ and 12‐month OS rates for the entire cohort were 51.6% and 32.7%, respectively, being 39.0% and 23.3% for patients with visceral metastases and 55.7% and 35.8%, respectively, for patients without visceral metastases. Figure shows the Kaplan–Meier survival of the whole group, and the subgroups with and without visceral metastases.

Compared with patients without visceral metastases, those with visceral metastases had significantly poorer performance status (eight [19.0%] vs. 11 [8.6%] had KPS <50, *P* < 0.044) and lower preoperative serum albumin concentrations (10 [35.7%] vs. seven [8.2%] had <35 g/L, *P* < 0.001). There was no statistically significant difference in any of the following characteristics between the two groups: age (*P* = 0.733), sex (*P* = 0.423), preoperative and postoperative Frankel scores (*P* = 0.437 and 0.507), other bone metastases (*P* = 0.292), postoperative KPS (*P* = 0.384), number of involved vertebrae (*P* = 0.826), pathological fracture (*P* = 0.720), metastasis site (*P* = 0.788), sphincter dysfunction (*P* = 0.877), interval between developing motor deficits and surgery (*P* = 0.466), change in Frankel grade (*P* = 0.414), and KPS (*P* = 0.306) (Table 1).

### *Overall Prognostic Factors*

Univariate analysis by the Kaplan–Meier method and log‐rank test identified the following significant prognostic factors for OS: rate of growth of primary tumor (*P* = 0.015), preoperative Frankel grade (*P* < 0.001) and KPS (*P* < 0.001), change in Frankel grade (*P* < 0.001) and KPS (*P* < 0.001), adjuvant therapy (*P* = 0.001), location of metastases (*P* = 0.021), and local relapse (*P* = 0.020) (Table 2). However, multivariable analysis with maximal model identified rate of growth of primary tumor (*P* = 0.003), preoperative KPS (*P* < 0.001), change in KPS (*P* < 0.001), and Frankel grade (*P* = 0.091) as independent prognostic factors (Table 3).

**Table 2:** Results of univariate analysis by log‐rank test

Variables	Whole cohort (%)		Median (m)	*P* value	Patients with visceral metastasis (%)		Median (m)	*P* value	Patients without visceral metastasis (%)		Median (m)	*P* value
	6 m	12 m			6 m	12 m			6 m	12 m		
Type of primary tumor by growth speed												
Group A (Rapid)	41.1	26.5	6.0 ± 0.5	**0.015** *	21.4	21.4	3.0 ± 0.9	0.074	45.6	27.7	6.0 ± 0.7	**0.041** *
Group B (Moderate)	54.9	32.6	7.0 ± 1.0		47.4	16.8	5.0 ± 1.5		58.1	38.6	8.0 ± 1.6	
Group C (slow)	73.1	50.0	10.0 ± 3.5		55.6	44.4	5.0 ± 1.9		82.4	52.9	13.0 ± 4.8	
Gender												
Male	59.8	36.3	6.0 ± 0.7	0.547	46.2	38.5	4.0 ± 0.6	0.250	50.9	31.7	7.0 ± 0.6	0.864
Female	46.2	30.3	8.0 ± 1.1		37.9	19.7	5.0 ± 1.1		62.7	41.2	9.0 ± 1.6	
Age												
≥65	48.6	32.6	6.0 ± 1.1	0.931	30.4	26.1	5.0 ± 2.2	0.273	49.3	31.4	6.0 ± 1.0	0.370
<65	53.2	32.6	7.0 ± 0.6		52.6	25.3	5.0 ± 0.5		58.9	37.5	8.0 ± 1.0	
Systematic comorbidity												
Yes	46.4	32.9	6.0 ± 0.9	0.721	46.2	23.1	5.0 ± 1.8	0.630	46.5	36.4	6.0 ± 0.8	0.995
No	54.3	32.6	7.0 ± 0.7		35.7	24.5	5.0 ± 0.6		60.6	35.5	8.0 ± 0.9	
Visceral metastasis												
Yes	39.0	23.3	5.0 ± 0.5	0.080	‐	‐	‐	‐	‐	‐	‐	‐
No	55.7	35.8	7.0 ± 0.8		‐	‐	‐		‐	‐	‐	
Preoperative Frankel grade												
A‐C	42.4	13.8	5.0 ± 1.3	**<0.001** *	27.3	9.1	4.0 ± 0.8	**0.035** *	49.0	17.8	6.0 ± 1.7	**0.006** *
D‐E	54.2	37.6	7.0 ± 0.8		45.2	31.5	5.0 ± 0.9		57.0	39.6	7.0 ± 1.4	
Preoperative KPS												
80–100	58.3	43.3	8.0 ± 2.1	**<0.001** *	41.2	29.4	5.0 ± 0.8	0.240	65.0	48.2	12.0 ± 3.5	**<0.001** *
50–70	53.9	32.0	7.0 ± 0.7		47.1	26.5	4.0 ± 1.3		55.9	33.9	7.0 ± 0.9	
10–40	21.1	0.0	3.0 ± 0.4		25.0	12.5	4.0 ± 0.7		18.2	0.0	3.0 ± 0.4	
Extrospinal bone metastasis												
Yes	49.0	34.2	6.0 ± 0.8	0.234	36.4	27.3	5.0 ± 0.9	0.466	54.3	37.4	7.0 ± 1.2	0.437
No	53.7	31.5	7.0 ± 0.7		45.0	24.0	5.0 ± 0.7		56.2	33.6	7.0 ± 1.1	
Pathological fracture												
Yes	61.4	39.9	9.0 ± 2.1	0.297	44.4	0.0	4.0 ± 0.5	0.868	68.2	48.7	10.0 ± 3.7	0.224
No	49.3	31.0	6.0 ± 0.5		39.4	27.3	5.0 ± 0.7		52.5	32.2	7.0 ± 0.5	
Number of involved vertebrae												
Solitary	54.8	34.8	6.0 ± 0.6	0.075	40.0	24.0	5.0 ± 0.5	0.791	52.6	30.3	7.0 ± 0.7	0.253
Multiple	56.8	38.5	7.0 ± 1.0		40.9	26.5	5.0 ± 0.9		57.8	39.5	8.0 ± 1.7	
Time to developing motor deficit												
≤5 days	48.2	33.1	6.0 ± 0.5	0.370	37.5	30.1	5.0 ± 1.6	0.549	52.0	34.1	7.0 ± 0.5	0.159
>5 days	60.8	31.4	8.0 ± 1.0		50.0	10.0	5.0 ± 0.6		64.0	38.1	9.0 ± 1.6	
Urinary retention/incontinence												
Yes	38.5	15.4	4.0 ± 0.9	0.131	33.3	0.0	2.0	0.078	40.0	20.0	4.0 ± 1.6	0.322
No	52.7	34.2	7.0 ± 0.6		41.0	27.2	5.0 ± 0.5		56.7	36.6	7.0 ± 0.8	
Serum album level pre‐operation												
≥35g/L	55.2	33.5	7.0 ± 0.9	0.300	43.8	27.1	4.0 ± 0.8	0.289	55.2	35.9	7.0 ± 1.1	0.791
<35g/L	44.4	27.8	4.0 ± 1.0		30.0	20.0	4.0 ± 0.8		57.1	28.6	7.0 ± 1.8	
Adjuvant therapy												
Yes	59.3	33.7	8.0 ± 0.8	**0.001** *	50.0	32.8	6.0 ± 1.4	0.090	63.3	41.2	9.0 ± 1.3	**<0.001** *
No	29.0	17.8	5.0 ± 0.6		27.8	16.7	4.0 ± 0.8		24.4	11.2	5.0 ± 0.6	
Location of involved vertebrae												
Cervical	66.8	50.9	14.0 ± 7.0	**0.021** *	33.3	33.3	3.0	0.295	80.4	57.5	18.0 ± 6.5	**0.043** *
Non‐cervical	49.3	39.1	6.0 ± 0.6		41.7	23.6	5.0 ± 0.6		51.9	32.3	7.0 ± 0.7	
Change on Frankel grade												
Deteriorated	13.3	6.7	3.0 ± 0.7	**<0.001** *	0.0	0.0	2.0	**0.013** *	15.4	7.7	5.0 ± 0.7	**<0.001** *
Not changed	36.0	26.1	5.0 ± 0.4		25.0	20.8	5.0 ± 0.5		41.2	28.8	6.0 ± 0.8	
Improved	74.4	44.3	10.0 ± 2.0		66.7	30.0	8.0 ± 2.4		76.6	47.6	10.0 ± 1.8	
Change on KPS												
Deteriorated	18.2	4.5	4.0 ± 0.7	**<0.001** *	20.0	0.0	3.0 ± 1.1	**<0.001** *	17.6	5.9	5.0 ± 1.0	**<0.001** *
Not change	26.7	10.2	5.0 ± 0.4		0.0	0.0	4.0 ± 0.4		38.8	14.8	6.0 ± 0.9	
Improved	70.5	49.4	12.0 ± 1.7		68.2	43.4	9.0 ± 3.4		71.1	51.1	13.0 ± 1.8	
Local relapse												
Yes	70.0	52.1	14.0 ± 3.1	**0.020** *	66.7	66.7	14.0 ± 9.0	**0.020** *	70.8	48.7	10.0 ± 3.9	0.261
No	47.5	28.3	6.0 ± 0.5		34.3	15.7	5.0 ± 0.5		52.1	32.6	7.0 ± 0.5	
Major complications												
Yes	40.0	26.7	4.0 ± 1.5	0.823	0.0	0.0	2.0 ± 0.0	**<0.001** *	50.0	33.3	6.0 ± 2.6	0.492
No	52.7	33.2	7.0 ± 0.6		42.1	25.1	5.0 ± 0.5		56.3	36.0	7.0 ± 0.8	

Note: *, statistical significance; KPS, Karnofsky performance status; m, months; −, not included.

**Table 3:** Significant prognostic factors according to multivariate analysis by Cox hazard proportional model

Prognostic factors	Hazard ratio	95% confidence interval	*P* value
**Whole cohort**			
**Primary tumor**			
Group C(slow)	1	‐	0.003
Group B (moderate)	1.76	1.05–2.95	0.032
Group A (rapid)	2.43	1.45–4.09	0.001
**Preoperative KPS**			
80–100	1	‐	<0.001
50–70	1.66	1.08–2.53	0.020
10–40	3.72	2.05–6.76	<0.001
**Change on KPS**			
Improved	1	‐	<0.001
Not change	2.62	1.69–4.04	<0.001
Deteriorated	4.26	1.98–9.17	<0.001
**Change on Frankel grade**			
Improved	1	‐	0.091
Not changed	1.58	1.02–2.45	0.043
Deteriorated	1.84	0.76–4.46	0.179
**Patients with visceral metastasis**			
**Change on KPS**			
Improved	1	‐	0.001
Not change	2.39	1.45–3.92	0.037
Deteriorated	3.12	1.07–9.09	0.001
**Major complications**			
No	1	‐	0.003
Yes	11.59	2.27–59.17	
**Patients without visceral metastasis**			
**Primary tumor**			
Group C (Slow)	1	‐	0.016
Group B (Moderate)	1.67	0.88–3.17	0.116
Group A (Rapid)	2.37	1.29–4.37	0.005
**Change on KPS**			
Improved	1	‐	0.001
Not change	2.45	1.36–3.71	0.002
Deteriorated	3.69	1.26–10.80	0.017
**Preoperative KPS**			
80–100	1	‐	<0.001
50–70	1.93	1.17–3.19	0.010
10–40	6.72	3.12–14.50	<0.001

Note: *P* < 0.05 was considered to denote a significant difference; KPS, Karnofsky performance status.

### *Prognostic Factors in Patients with Visceral Metastases*

Preoperative Frankel score (*P* = 0.035), change in Frankel grade (*P* = 0.013) and KPS (*P* < 0.001), local relapse (*P* = 0.020), and major complications (*P* < 0.001) were potential prognostic factors according to univariate log‐rank test (Table 2). The multivariate Cox regression model identified change in KPS (*P* = 0.001) and major complications (*P* = 0.003) as the only variables that were independent predictors of OS (Table 3).

### *Prognostic Factors in Patients without Visceral Metastases*

Univariate analysis identified the potential prognostic factors of rate of growth of primary tumor (*P* = 0.041), preoperative Frankel score (*P* = 0.006) and KPS (*P* < 0.001), adjuvant therapy (*P* < 0.001), change in Frankel grade (*P* < 0.001) and KPS (*P* < 0.001), and location of metastases (*P* = 0.043) (Table 2). The multivariate Cox regression model showed that primary tumor (*P* = 0.016), change in KPS (*P* = 0.001), and preoperative KPS (*P* < 0.001) had significant influence on OS (Table 3).

## Discussion

Currently, most published studies that have focused on assessing prognostic factors in patients with MSCC have failed to distinguish between patients with and without visceral metastases. To the best of our knowledge, this is the first study to investigate the impact of visceral metastases on OS and identify different prognostic factors according to visceral metastases status.

A randomized controlled study^10^ and a meta‐analysis^22^ have found that surgery is superior to radiotherapy alone in terms of functional outcome, pain control, and OS. However, not all patients with MSCC benefit from undergoing a surgical procedure. Especially in patients with short survival times, post‐operative complications may offset the intended benefits of surgery, or death may occur before wound healing or functional recovery.

In general, patients with very short survival times are not suitable candidates for decompressive surgery^10^. Therefore, means of accurately predicting survival time in patients with MSCC is currently an important topic to research. Various prognostic scoring systems for predicting life expectancy of patients with MSCC have been developed. The scoring systems reported by Tomita^12^ and Tokuhashi^13^, ^14^ are the most representative and commonly used systems; both use visceral metastases as an important prognostic factor for survival in these patients.

### *Effect of Visceral Metastases on Prognosis*

Understandably, development of visceral metastases, an indicator of more aggressive tumors, is usually regarded as denoting an advanced stage of cancer. Patients with visceral metastases tend to have shorter survival because of cancer progression^3^. Lei *et al.* 23 reported that visceral metastases have a significant impact on survival in patients with MSCC from lung cancer. Crnalic *et al.* 24 observed that visceral metastases have a detrimental effect on survival of patients with prostate cancer, the median survival of patients with visceral metastases being only 4 months, as compared twitho 10 months for patients without visceral metastases. Drzymalski *et al.* 25 found that the presence of additional metastases at the time of diagnosis of spinal metastases is independently associated with a shorter overall survival.

Surprisingly, our results differed substantially from those previously reported using the scoring systems of Tokuhashi^13^, ^14^ and Tomita^12^. Our results were conflicting in that patients with visceral metastases did not have a significantly shorter survival time than those with spinal metastases alone. However, this finding was in accordance with other previous reports. Sellin^26^ reported that visceral metastases do not affect prognosis according to multivariate analysis, their univariate analysis showed that it was significantly associated with worse overall survival. Jiang^27^ identified no significant effect of the absence or presence of visceral metastases on postoperative recurrence or survival. In another study by Sciubba^28^, the median survival of patients without visceral metastases was 28.0 months, compared with 17.4 months for those with visceral metastases. However, the results of our multivariate analysis were similar to those of Arrigo^20^ and Chong^21^ in showing no statistically significant difference between patients with versus without visceral metastases.

In addition, visceral metastases status reportedly has a similar impact on prognosis in patients with different primary tumor types. Zadnik^29^ examined the relationship of visceral metastases to survival in patients with MSCC from breast cancer and found that the median survival for those without visceral metastases was 25.9 months, compared with 28.1 months for those with visceral metastases; this difference was not significant on Mantel‐Cox testing. Chen^30^ reported that visceral metastases had no statistically significant association with survival in patients with non‐small‐cell lung cancer and spinal metastases who underwent spinal surgery. The findings of Park et al. were similar^31^. In addition, Ju^32^ demonstrated that visceral metastases had no statistically significant association with survival in patients with MSCC from prostate cancer and Bakker^33^ found that they were not significantly associated with survival in patients with renal cell carcinoma. Walcott^34^ found that the concomitant presence of visceral lesions or multi‐focal bony disease did not have prognostic significance in patients with breast cancer. Thus, the presence of progressive systemic disease should not be a contradiction to aggressive surgery, which is in agreement with previous reports by Walcott *et al.* 34.

The explanation for our results is unclear. One possible explanation is that the presence of spinal metastases in itself denotes a more aggressive and advanced stage of cancer than the presence of visceral metastases. Thus, survival is equivalent for patients with and without visceral metastases. Another possible explanation is that advanced treatment strategies, such as targeted therapy, hormonal therapy, chemotherapy and stereotactic body radiotherapy, effectively control systemic metastases and significantly prolong the survival time of patients with MSCC. It is also possible that there was a bias in selecting patients for surgery, because patients with visceral metastasis usually have lower performance scale scores, which can be considered a contraindication for surgery. Additionally, differences in stage at diagnosis may have influenced our results. Another possibility is that visceral metastases may not affect the prognosis of certain types of primary cancer and those types may have accounted for a larger proportion of our study cohort, which may in turn have influenced our results.

### *Difference in Prognosis between Patients with and without Visceral Metastases*

In the current study, we did not find a correlation between the presence of visceral metastases and decreased survival. However, we found to our surprise that patients with and without visceral metastases have different prognostic factors. The factors influencing survival times of patients with visceral metastases were change on KPS and postoperative complications, whereas rate of growth of primary tumor, pre‐operative KPS, and change in KPS were significantly associated with survival in patients without visceral metastases. These findings are in agreement with previous reports that have demonstrated that KPS, neurological compromise, and primary cancer type are associated with decreased survival. However, we did no further analysis to determine why and how other prognostic factors affect survival, because we only aimed to investigate the correlation between visceral metastases and survival. Of course, we believe that identifying these prognostic differences is important for selecting optimal treatment.

### *Primary Tumor*

The prognostic impact of type of primary tumor on survival of patients with MSCC has been reported previously^12^, ^13^, ^14^. Favorable histologic types such as breast and prostate cancer are associated with better survival prognosis than other types, whereas survival of patients with lung cancer is extraordinarily poor^20^, ^35^. In our study, the rate of growth of the primary tumor was a significant prognostic factor in the whole group and the group without visceral metastases. However, we did not determine whether the type of primary tumor influences survival of patients with visceral metastases.

### *KPS or Change in KPS*

In our study, change in KPS 4 weeks postoperatively was a significant prognostic factor in the whole cohort, as well as in both groups with and without visceral metastases. Preoperative KPS was a significant prognostic factor in the whole cohort and the group without visceral metastases, but not in the group with visceral metastases. One possible explanation is that poor preoperative KPS, or no or worsening KPS, denotes a more aggressive cancer or more advanced stage. Additionally, major complications may affect the prognosis of patients with visceral metastases. However, few studies have reported the prognostic impact of complications in patients with MSCC. In our study, the findings concerning influence of complications on prognosis may be questionable because of the large difference in the number of patients with or without complications (15 vs. 154). Further study is required to better address this question.

### *Limitations of the Study*

First, this was a retrospective review with a small number of patients with visceral metastases, the small number possibly being attributable to financial considerations and the negative attitude of Chinese people toward seeking surgical treatment, especially for patients with visceral metastases. Second, a wide variety of primary tumors were included and different tumor types may have different biological behavior and different prognoses. It would likely be useful to analyze prognosis for individual tumor types rather than grouping all tumor types together. However, Abouret *et al.* 36 reported similar results in that they found that visceral metastases were not significantly predictive of long‐term survival for various primary tumors. Third, in the present study we did not investigate the effect of chemotherapy because previous chemotherapy regimens varied between patients; those variations may have influenced survival. Last, we found it difficult to decrease heterogeneity between the two groups. Nonetheless, we believe that our findings are valid. Additionally, selection bias is inevitable in retrospective cohort studies^37^.

In summary, visceral metastases had no statistically significant association with survival in patients with MSCC; thus, more aggressive surgery should be considered for patients with visceral metastases.
